# Intersection of Health Informatics Tools and Community Engagement in Health-Related Research to Reduce Health Inequities: Scoping Review

**DOI:** 10.2196/30062

**Published:** 2021-11-19

**Authors:** Geetanjali Rajamani, Patricia Rodriguez Espinosa, Lisa G Rosas

**Affiliations:** 1 Department of Human Biology Stanford University Stanford, CA United States; 2 Department of Epidemiology and Population Health School of Medicine Stanford University Stanford, CA United States

**Keywords:** community engagement, stakeholder involvement, underserved communities, health informatics, health information technology, health inequities, health-related research

## Abstract

**Background:**

The exponential growth of health information technology has the potential to facilitate community engagement in research. However, little is known about the use of health information technology in community-engaged research, such as which types of health information technology are used, which populations are engaged, and what are the research outcomes.

**Objective:**

The objectives of this scoping review were to examine studies that used health information technology for community engagement and to assess (1) the types of populations, (2) community engagement strategies, (3) types of health information technology tools, and (4) outcomes of interest.

**Methods:**

We searched PubMed and PCORI Literature Explorer using terms related to health information technology, health informatics, community engagement, and stakeholder involvement. This search process yielded 967 papers for screening. After inclusion and exclusion criteria were applied, a total of 37 papers were analyzed for key themes and for approaches relevant to health information technology and community engagement research.

**Results:**

This analysis revealed that the communities engaged were generally underrepresented populations in health-related research, including racial or ethnic minority communities such as Black/African American, American Indian/Alaska Native, Latino ethnicity, and communities from low socioeconomic backgrounds. The studies focused on various age groups, ranging from preschoolers to older adults. The studies were also geographically spread across the United States and the world. Community engagement strategies included collaborative development of health information technology tools and partnerships to promote use (encompassing collaborative development, use of community advisory boards, and focus groups for eliciting information needs) and use of health information technology to engage communities in research (eg, through citizen science). The types of technology varied across studies, with mobile or tablet-based apps being the most common platform. Outcomes measured included eliciting user needs and requirements, assessing health information technology tools and prototypes with participants, measuring knowledge, and advocating for community change.

**Conclusions:**

This study illustrates the current landscape at the intersection of health information technology tools and community-engaged research approaches. It highlights studies in which various community-engaged research approaches were used to design culturally centered health information technology tools, to promote health information technology uptake, or for engagement in health research and advocacy. Our findings can serve as a platform for generating future research upon which to expand the scope of health information technology tools and their use for meaningful stakeholder engagement. Studies that incorporate community context and needs have a greater chance of cocreating culturally centered health information technology tools and better knowledge to promote action and improve health outcomes.

## Introduction

The field of health informatics is defined as “the science of how to use data, information, and knowledge to improve human health and the delivery of health care services [1].” Health information technology—a vital part of informatics—refers specifically to electronic systems that are used to collect, store, share, and analyze health information [2]. Some common examples of health information technology include the electronic health records used widely across health systems, patient mobile apps for disease management, and websites with health information. Health information technology has been at the forefront of many national initiatives, highlighting its potential to improve health care quality, increase patient safety, enhance communications and patient outcomes, and reduce health care costs. With the exponential growth in health information technology, various tools are being explored and increasingly utilized to facilitate patient engagement in health-related research, with tips and best practices starting to emerge [2]. These tools mostly target individual patient engagement, for example, the electronic patient portal and apps for disease management [3].

A key opportunity lies in using health information technology to engage communities and stakeholders from multiple sectors in health-related research as a potentially powerful method of involving traditionally underrepresented groups in research and promoting health equity. The importance of involving patients and other stakeholders in research is underscored by program initiatives such as the Patient-Centered Outcomes Research Institute (PCORI) [4]. A growing evidence base points to enhanced recruitment, retention, and relevance of research questions if relevant stakeholders are engaged [5].

Community engagement presents a key opportunity to bring additional knowledge and lived experience to social determinants of health, or the conditions in which we live, work, and play (including internet access), which are increasingly recognized as key drivers of health outcomes [6]. Community-engaged research is a promising strategy for addressing social determinants of health and health inequities [7]. Community-engaged research entails working collaboratively with groups of people associated with each other by geography, special interest, or similar situations to address relevant issues affecting their health and well-being [8]. Health information technology can be utilized to facilitate this community engagement and stakeholder participation. Research involving the intersection of community-engaged research and health information technology or health informatics is emerging and beginning to lay a foundation for the generation of supporting evidence. Current applications range from digital tools for participant recruitment, to social media and infographics for delivering health messages to participatory design, development, and deployment of disease-specific management tools such as apps and websites [9-11].

Current health information technology literature is predominantly focused on individual patient engagement strategies in health care [5]. However, increasingly, health information technology is being employed to engage patients and communities in health research. As the research around health information technology and community-engaged research grows, there is a need to build a body of evidence to highlight success stories, potential challenges and lessons learned, and stimulate future directions in this area of inquiry. We conceptualize the intersection of health information technology and community-engaged research as the meaningful involvement of community members or relevant stakeholders in either or both of the following: (1) the design of health information technology or adaptation of existing tools for cultural appropriateness and effectiveness, and (2) utilization of health information technology tools in community-engaged research studies. With this conceptualization in mind, we aimed to (1) conduct a scoping review of existing literature to fill in the knowledge gap around populations, community engagement strategies, health information technology tools, and outcomes in research at the intersection of health information technology and community-engaged research, and (2) lay the foundation to promote additional ideas to expand the breadth and depth of technology utilization for community and stakeholder engagement.

## Methods

We followed the 5-step framework developed by Arksey and O’Malley [12] and updated by Levac et al [13]. This comprised (1) identifying the research question; (2) identifying relevant studies; (3) study selection; (4) charting data; (5) summarizing and reporting the results.

### Step 1: Identifying the Research Question

The literature review was conducted to answer the following 4 research questions about health information technology and community-engaged research: (1) What types of populations were included in these studies? (2) What community engagement strategies were utilized? (3) What types of health information technology tools were used? and (4) What outcomes of interest were measured?

### Step 2: Identifying Relevant Studies

A search strategy was developed with the following inclusion criteria: (1) English-language papers; (2) published between 2010 and 2019; (3) peer-reviewed papers only; (4) any geographic location; and (5) search term found in title or abstract. We searched 2 key databases in summer 2019: PubMed and PCORI Engagement in Health Research Literature Explorer. The searches were conducted using various combinations of the following words and phrases: “health information technology,” “technology,” “health informatics,” “informatics,” “eHealth,” “mHealth,” “health,” “community engagement,” “community participation,” “community involvement,” “community-engaged research,” “stakeholder involvement,” and “citizen science.” Note that search terms were intentionally kept broad to capture any work being done in this space; specific types of communities engaged (eg, racial or ethnic groups, LGBTQ populations, etc) were included as long as the studies involved some form of health information technology and community-engaged research.

### Step 3: Study Selection

After screening papers for duplicates, titles and abstracts were screened for relevance to the topic (ie, community engagement and health information technology). Examples of papers excluded for lack of topic alignment were studies with key words about community participation but that did not entail community engagement (eg, studies enrolling community participants to complete surveys without any participatory engagement of community members or stakeholders in the research process). The next largest exclusion category was studies that used a citizen science approaches, but without a human health or health information technology focus (eg, studies on environmental health in which participants collect air or water quality data in a crowdsourcing approach that is not necessarily in a community engaged manner or influencing any research decision making). After this step, full texts were further screened to assess for eligibility.

### Step 4: Charting Data

The final papers were analyzed and coded using following criteria: (1) the population of interest (age, gender, race/ethnicity, geographic location, other relevant info such as socioeconomic status, sample number); (2) research objective or stated project aims; (3) health information technology tools used (eg, social media, SMS text messaging, web sites, other); (4) methods for community engagement (eg, community advisory boards, town halls, and designing health information technology tools collaboratively); and (5) outcomes of interest (eg, change in participants’ knowledge on topic, usability or update of websites, input on prototypes, advocacy for policy change). A few papers were initially reviewed by all authors to establish a model for data extraction and to discuss any relevant issues. Data extraction was then conducted primarily by the first author (GR), using Excel (Microsoft Office 365) and Word (Microsoft Office 365) software, and any cases that lacked clarity were resolved with input from coauthors (PRE or LGR). All analyses were double checked by coauthors before interpretation.

### Step 5: Summarizing and Reporting Results

Extracted data were summarized. Findings were synthesized to address the research questions, and figures and tables are used to present the results. The key takeaway messages from this review are presented below, along with identified gaps to be addressed in future research.

## Results

### Search Process Results

**Figure 1:**
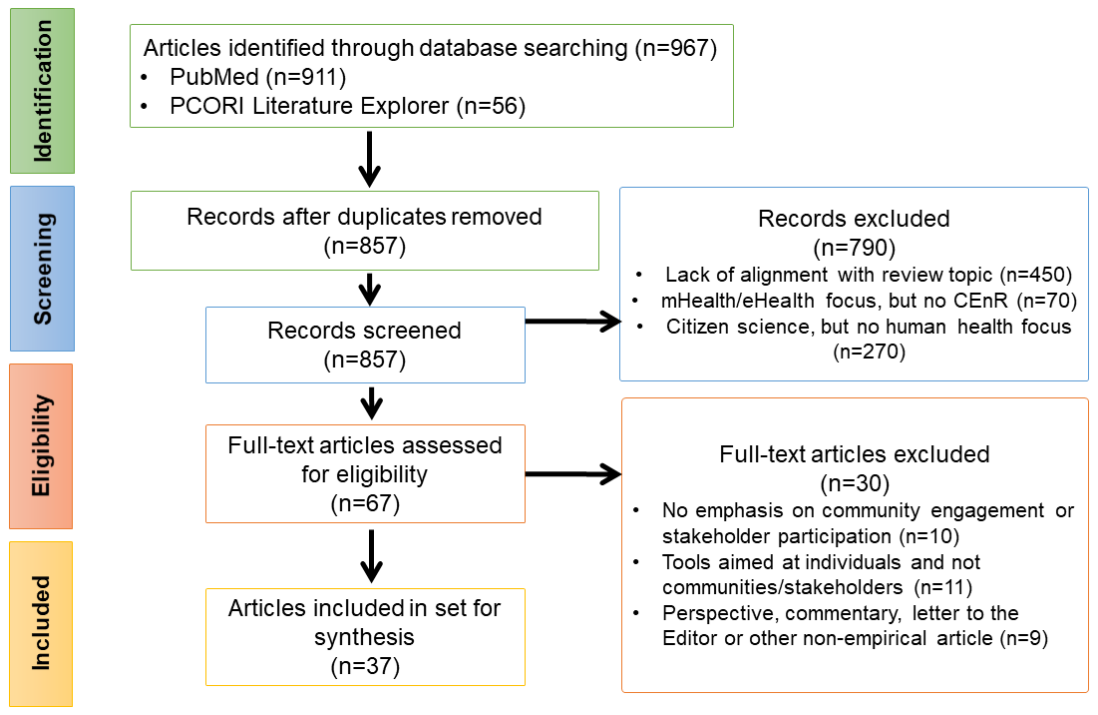
Screening process. CEnR: community-engaged research; PCORI: Patient-Centered Outcomes Research Institute.

A PRISMA (Preferred Reporting Items for Systematic Reviews and Meta-Analyses [14]) diagram shows the screening process (Figure 1). The search process yielded a total of 967 papers for screening—specifically, 911 papers from PubMed and 56 papers from the PCORI Literature Explorer. After removing duplicates and performing title and abstract screening, a total of 67 papers remained. Full-text screening of these papers resulted in 30 papers being excluded; therefore, 37 remained and were included in this review. The number of papers in this area have increased over time. Of the 37 papers included, only 13 were from the first 5 years of the time period (2010-2014), while 24 of the papers were from the period 2015-2019.

### Types of Populations Represented

The communities engaged were generally populations that have been underrepresented in health-related research, including racial or ethnic minority communities, such as Black/African American, American Indian/Alaska Native, Latino ethnicity, and communities with low socioeconomic status (Table 1). The studies focused on various age groups, such as K-12 (for designing apps for healthy snacking [15]), teens (for participatory design of a website around sexually transmitted infections [16]), and older adults (for soliciting input around creating active lifestyle/supportive neighborhoods [17]). Study populations were also geographically spread across the United States, and a few studies were conducted in other countries.

**Table 1:** Population demographics by community engagement strategies.

	Demographic details	Study location	Reference
**Collaborative health information technology development and promotion**			
	**Collaborative health information technology development**		
	K-12 children, Low socioeconomic status	Colorado	[10]
	K-12 children, Low socioeconomic status	Colorado	[15]
	>18 years, Varying health literacy	Washington Heights/Inwood region, New York City	[18]
	Providers affiliated with a major health system	Southeast Minnesota	[19]
	People of color	Harlem, New York	[20]
	>18 years, Varying levels of health literacy	New York	[21]
	18-29 years	San Francisco Bay Area	[22]
	Adults with serious mental illnesses	Massachusetts	[23]
	Median age 27 years, Sexual (98%) and gender (15%) minority	—^a^	[24]
	Gender minority (n=3813)	Across the United States	[25]
	**Health information technology engagement through community advisory boards or other techniques^b^**		
	Pregnant women with type 1 diabetes	Sweden	[26]
	Stakeholders and clinicians	University of Washington	[27]
	Stakeholders and health care providers	Netherlands	[28]
	Racial minority (90%); low socioeconomic status	New York	[29]
	58% to 200% of the federal poverty level	Chicago	[30]
	Addresses a spectrum of underrepresented minorities	—	[31]
	Black/African American; resource-limited neighborhood	Washington, D.C.	[32]
	Young adults and teenagers, Black/African American	—	[33]
	**Information needs for health information technology through focus groups**		
	Parents of children <8 years, Hispanic; low socioeconomic status	New Mexico	[34]
	Mean 18.4 years; 66% female , 80% Black/African American	—	[11]
	Patients with end-stage renal disease	Baltimore, Maryland	[35]
	14-24 years; 71% female, Black/African American	Michigan	[16]
	American Indian/Alaska Native College students	Kansas, Missouri	[36]
	>50 years, Women; Black/African American; with HIV^c^	Baltimore-Washington metropolitan area	[37]
	Marginalized communities (patients with history of incarceration)	—	[38]
	51-74 years, Black/African American women; high cardiovascular disease	Washington, DC	[39]
**Health information technology to engage communities in research**			
	**Health information technology apps for research and advocacy using a citizen science approach**		
	>65 years; 75% female, Low socioeconomic status homes for older adults	San Mateo County	[40]
	Mean 64 years	4 rural communities in New York	[41]
	50+ years	Haifa, Israel	[17]
	Adults and adolescents, Hispanic (low- or middle-income country)	Mexico	[42]
	Adolescents and older adults, Latino; low socioeconomic status	North Fair Oaks, California	[43]
	>18 years, Racially or ethnically diverse; low socioeconomic status; food insecure	San Mateo	[44]
	>18 years, High rates of poverty or unemployment	Camden, New Jersey	[45]
	>18 years, Chronic stress environment	Bay Area, California	[46]
	>65 years	Queensland, Australia	[47]
	Multi-ethnic community residents from diverse socioeconomic backgrounds	Bogota, Colombia; San Francisco, United States; and Temuco, Chile	[48]
	Elementary and middle school students, Low-density areas	Santa Clara	[49]

^a^Data not available.

^b^Other techniques included person-centered web support, community engagement and stakeholder-focused interviews, and clinic-community linkages

^c^HIV: human immunodeficiency virus.

### Community Engagement Strategies Utilized

Two approaches to community engagement emerged. First, some researchers employed community engagement in studies for collaborative health information technology development and promotion. These included collaborative development and partnerships to promote health information technology use; health information technology engagement through community advisory boards and other techniques; and focus groups to assess community health information needs. The next approach involved using health information technology to engage communities in participatory research related to questions beyond the use or development of the health technology. One key example of this participatory approach is the “citizen science” method whereby citizens (ie, community residents) participate as scientists and use health information technology tools for specific data collection and advocacy efforts.

### Collaborative Health Information Technology Development and Promotion

Collaborative development of health information technology and partnerships to promote health information technology use was the largest category of community engagement [10,15,18-25]. For instance, a study by Khan and colleagues [15] designed 4 mobile apps to promote healthy eating habits for children in low socioeconomic status households in a Boulder, Colorado neighborhood with high rates of poverty and chronic disease. Before testing their intervention, the study team built rapport with the community by tutoring children for over 100 hours, and additionally, they paired up with teenagers in the neighborhood in order to teach them how to use the app [15]. Smith et al [20] created a website for racial and ethnic minorities in Harlem, New York—a group with a disproportionately high prevalence of diabetes, cardiovascular disease, and other chronic conditions—to provide convenient access to health information, while tailoring content to those with low health literacy. Erguera et al [22] engaged youth and young adults (age 18-29 years), who share a disproportionate burden of the disease, to collaboratively develop a mobile app to improve medication adherence and HIV care in order to reduce the disease burden in this group. Community-engaged research was used to develop more useful and culturally relevant health information technology.

Several of the studies [26-33] in this category involved engaging community members to serve on advisory boards or councils. This approach ensures that community feedback is incorporated in various stages of the health information technology research process and that proposed tools or interventions meet the community’s needs. For instance, in one study [32], a community advisory board was established to evaluate physical activity monitoring technologies as one of its initiatives in resource-limited communities, and the community was engaged throughout the research process. Similarly, in the Moyo Health Network project [33], a Community Coalition Board was used to recruit young adults and launch an e-cohort to examine the factors for developing cardiovascular health inequities, as an innovative way to engage a younger population that often does not receive direct benefits from technology development (eg, health tech apps). Through iterative feedback, this community-engaged research approach engaged this group to improve the mHealth tool and thus resulted in a unique and tailored health information technology tool [33]; the cohort was linked by mobile phones to an open-source platform for collecting and processing data in a Big Data cloud environment. Moreover, the youth received internships and in-depth training in the tech sector to improve their economic and career prospects, which is a key community need.

Focus groups were also employed to solicit community input in the development of health information technology [11,16,34-39]. One study [34] designed a website for parents of Latino ethnicity and children from low socioeconomic status households, to educate them about mental health symptoms in children. Before designing the website, the research team engaged parents in this community via focus groups to elicit feedback and provided them with training on how to use the website after its creation. Another study [36] designed a culturally appropriate website for American Indian/Alaska Native college students about smoking and other health issues that affect this community by soliciting input on needs from students in the community. Wang et al [38] used community-engaged research and health information technology innovatively to engage stakeholders and improve health outcomes and care for formerly incarcerated individuals; data from clinical programs serving patients with histories of incarceration were leveraged to create a deidentified database with embedded web analytic tools. This engages stakeholders—including patients, clinicians, and policy makers—to access these data to understand the impact of incarceration. Importantly, it engages them in the research process to improve health outcomes.

### Health Information Technology to Engage Communities in Participatory Research

The second set of studies [17,40-49] employed health information technology to engage community members in answering community-driven research questions. Many tapped into the power of citizen science by engaging the members of the community in research specific to areas that affect their community or neighborhood [50]. For instance, in the Our Voice global initiative [40], citizen scientists utilize an easy-to-use mobile app tool (Healthy Neighborhood Discovery Tool) and collect data pertinent to a relevant research question, engage in data analysis, contribute to discussion of findings in community meetings to develop and prioritize community driven solutions and finally advocate for change. This innovative model of citizen science–driven community engagement using a health information technology tool was a predominant model found in many studies included in our review. Studies used the Our Voice model to classify neighborhood elements for active living in various locations across the globe [17,40,42,43,47,48], study specific situations such as enhancing safe routes to school [49], understand assets or barriers to healthy eating and active living in rural settings [41], and identify healthy food access [44,45] and neighborhood stressors [46].

### Extent of Community Engagement at Various Stages of the Research Process

Studies were classified (Table 2) in the following categories based on their engagement of community members or stakeholders in the following stages of the research process: (1) contributing to research priorities; (2) offering feedback on health information technology designs or prototypes; (3) participant recruitment; (4) collecting data using the health information technology tool; (5) reviewing study results; and (6) advocacy and dissemination. The 2 most commonly used forms of engagement were engaging participants to offer feedback on a health information technology design or prototype (26 studies) and collecting data using the health information technology tool (21 studies). The 2 categories with the smallest number of studies involved community members contributing to research priorities (7 studies) and recruiting participants for research (6 studies). Classifications were not mutually exclusive, meaning any study could be classified into multiple categories based on the types of engagement used. Specifically, 27 of the 37 total studies analyzed were classified into multiple categories.

**Table 2:** Extent of community engagement.

Community engagement phase	Studies utilizing community engagement in this phase, n^a^	Reference
Contribute to research priority	7	[19,23,24,32-34,38]
Offer feedback on HIT^b^ design or prototype	26	[10,11,15,16,18-26,28-33,35-40,42]
Recruit participants for research	6	[11,25,30,32-34]
Collect data using HIT tool	21	[10,17,20,22,24,25,29,32-34,39-49]
Review study results	13	[17,25,37,40-49]
Engage in advocacy or dissemination	13	[17,19,27,40-49]

^a^Studies could be categorized into multiple categories.

^b^HIT: health information technology.

### Types of Health Information Technology Used

The types of technology used varied across different studies (Table 3), with mobile- or tablet-based apps as the most common platform used. Collaborative approaches involving stakeholders were commonly used to design mobile or tablet apps and comprise close to half of the studies assessed. This was followed by websites, comprising approximately 27% of studies. Creative or artistic approaches to community engagement such as infographics were also part of the studies analyzed. All studies paid special attention to using culturally appropriate technological interventions and involving participants in both the research design and evaluation.

**Table 3:** Technology interventions used in studies.

Type of technology	Studies utilizing this technology, n	Reference
Infographic	2	[18,21]
Data integration site	2	[19,38]
Sensor or multimedia	3	[10,32,35]
Data sharing portal	3	[25,27,29]
Website	10	[11,15,16,20,26,28,30,31,34,36]
Tablet or mobile app	17	[17,22-24,33,37,39-49]

### Outcomes of Interest

Overall, 4 main categories of outcomes emerged (Table 4): (1) elicit user needs and requirements, (2) assess and redesign health information technology tools and prototypes with participants, (3) measure knowledge, and (4) advocate for community change. Approximately one-third of the studies (13/37) fell under the assess and redesign health information technology tools and prototypes with participants category. The next largest category of studies aimed for advocacy for change in communities. Many of these studies were part of the global Our Voice citizen science-driven community engagement framework [17,40-50]. This was followed by community engagement for eliciting user needs and requirements related to design, development, and refinement of health information technology tools, as well as measuring knowledge. Evaluating impact was also of interest in many of the studies, regardless of their primary outcome.

**Table 4:** Outcomes of interest using health information technology supported community engagement techniques.

Category	Outcomes of interest
Elicit user needs and requirements	Youth use of information communication technologies, their communication about sexuality and HIV^a^/STIs^b^ [11] Design health intervention technologies for low socioeconomic status communities [15] Culturally appropriate informatics intervention for HIV/STI prevention [16] Describe stakeholder practices and challenges in genomic integration for personalized medicine [27] Guide for business modeling with stakeholder-oriented analysis for eHealth implementations [28] Provide support for pregnant diabetic women [26] Gather requirements for developing visuals and infographics [18] Inform development of HIV app for older adult women [37]
Assess health information technology tools and prototypes with participants	Mobile phone prototype for usability and usefulness in low socioeconomic status contexts [10] Website with tailored chronic illness content to consumers of color [20] Community engaged health informatics platform or website with participatory functionality [29] Develop tailored infographics to engage viewer and motivate healthy behaviors [21] Web analytics research platform to share data and engage in research [38] Culturally sensitive participatory approach app to increase intervention adoption [39] Acceptability of WYZ health app in youth living with HIV to improve HIV care [22] Effectiveness of mobile app to engage and recruit sexual and gender minority [24] Mobile app for adults with serious mental illnesses [23] Health information exchange and patient data portal for population health [19] Referral to community resources and system use [30] Feasibility of digital research platform to recruit & retain sexual and gender minority [25] Feasibility of participatory approach–monitoring wristbands in resource limited settings [32]
Measure knowledge	Participants’ knowledge of preschool children mental health [34] Perceptions and attitudes on internet use and health info needs for American Indian or Alaska Native individuals [36] Assess comprehension of renal replacement therapy knowledge [35] Promote future workforce training and mobile platform for health research [33]
Advocate for community change	Neighborhood elements that affect active living [40] Neighborhood barriers in low- or middle-income country settings [42] Barriers and solutions for food access and food behaviors [44] Elements that affect active living in a neighborhood in Australia [47] Healthy corner store network effectiveness for healthy food access [45] Elements of environment contributing to chronic stress [46] Effectiveness of Open Streets initiative to modify neighborhood [48] Feasibility of active and safe commuting to school [49] Elements affecting active living in rural settings [41] Elements affecting active living in an Israel city [17] Elements affecting active living in Latino neighborhood [43]

^a^HIV: human immunodeficiency virus.

^b^STIs: sexually transmitted infections.

## Discussion

### Key Findings

This scoping literature review showcases the current state of community and stakeholder engagement in health-related research that uses health information technology and how these 2 fields currently intersect. Collaborative development of health information technology tools and partnerships to promote use (encompassing collaborative development, use of community advisory boards, and focus groups for eliciting information needs) were the most frequent intersection between health information technology and community engagement. This was followed by use of health information technology to engage diverse communities in research (eg, through citizen science for social and environmental change). Overall, most studies targeted underrepresented communities, including racial or ethnic minority groups, and many included informational community and patient-facing tools, such as websites or educational apps. Mobile- and tablet-based apps were by far the largest category of health information technology tools used across studies, followed by websites. For most studies in this review, the concurrent use of community engagement and technology enhanced the cultural appropriateness of information or interventions regarding chronic diseases and other conditions that disproportionately affect underserved and underresourced communities.

This scoping review revealed that the number of research studies that utilize health information technology alongside community-engaged research is relatively limited, although increasing. This suggests room for expansion of future research involving various technologies for improving outcomes beyond the individual level (eg, at household or community level) and for meaningful engagement of communities and stakeholders from multiple sectors. This is particularly relevant as existing evidence indicates the presence of a digital divide, with racial or ethnic and other underserved communities having less access and familiarity with health information technology [51,52]. Our findings showcase the opportunity and ways in which the use of health information technology tools, along with a variety of community-engaged research approaches, can yield more meaningful outcomes and impacts. One example of the innovation and impact that result from combining health information technology and community-engaged research can be seen in the body of research conducted by the Our Voice global initiative [17,40-50], which utilized a novel app technology (developed using community-engaged research) to engage communities in relevant research with the ultimate goal of developing locally driven and feasible solutions to address a variety of real-world problems. This framework [40] thus represents a key model for the potential increase and current use of health information technology in community-engaged research. This body of research identified in this review showcases the power of health information technology for actively involving communities in health research, while also engaging them in advocacy efforts for change at multiple levels (eg, built environment, policy).

Our findings also highlight the power of using community-engaged research and health information technology to design interventions relevant to the specific populations of interest and their health priorities including: designing websites with chronic illness content tailored to racial minorities [20], investigating the feasibility of activity-monitoring wristbands in internet-limited neighborhoods [32], and designing apps to improve HIV care for youth and young adults [22]. In addition, community engagement has been used as an avenue to measure community knowledge about a topic of interest, such as renal replacement therapy [35] or the mental health of preschool children [34], which can then inform future interventions or other efforts. Finally, the variety of approaches used, which ranged from focus groups to community advisory boards to citizen science techniques, displays the range of the possibilities for community engagement approaches, as well as the range in the depth of stakeholder involvement in various aspects of the research—from engagement of community members at the start for needs assessment to engagement throughout the research process, including data collection, dissemination, and advocacy efforts.

This study furthers existing knowledge based on published literature reviews on patient engagement approaches in the health-related research context [53] and on improving outcomes of health information technology initiatives [5] and is unique in its focus on community engagement. Patient engagement in health research is typically used to refer to participation in the study (ie, research participation) as patient advisors [54]. There are few examples in the literature about meaningful engagement throughout the research process and how study protocols, processes, and outputs can significantly change after collaboration with community members and relevant stakeholders. Petersen [55] advocates for patient informaticians who proactively develop and implement technologies for better management of health and lifestyle. With the growth of participatory medicine [56] and shared decision-making, this review is timely and makes a contribution by highlighting the state of community-engaged research within the health information technology literature.

Finally, the intersection of community-engaged research and health information technology might look different today, given the COVID-19 pandemic and its impact on digital health technology. Since the conclusion of our search, we have seen a significant uptake in telehealth tools and virtual forms of engaging communities and stakeholders in response to the COVID-19 pandemic. For example, Peeters et al [57] created a global data-sharing initiative to understand COVID-19 severity in patients with multiple sclerosis and provide data-driven clinical insights. Relevant stakeholders were engaged to decide upon data sets, participate in study design, and create a user-friendly pipeline for sharing data at a global scale. During the pandemic, additional funding and attention, including from the National Institute of Health, has been provided for community engagement [58]. The use of health information technology, innovations, and areas in need of additional development and collaboration should be assessed. Future studies should also evaluate whether our findings are also valid during the pandemic and to assess potential new or innovative tools—such as that created by Peeters et al [57]—that have emerged in response to the pandemic.

### Strengths and Limitations

This review makes a unique contribution to the literature, with a focus on health information technology and community-engaged research, given that the majority of health information technology engagement strategies focus on individuals as end users rather than on patients as research partners and community engagement. We reviewed the body of literature in the last decade during which the field of health information technology–related research has been growing; hence, this review focuses on an important timeframe. By presenting research to-date and covering a breadth of information, this review also highlights gaps and points to opportunities, which can be utilized to shape future directions of community-engaged research supported by health information technology.

The search process was designed to be comprehensive, but it is possible that some search terms were omitted (eg, telehealth, participatory medicine). Additionally, it is possible that a wider range of results may have been obtained by searching additional databases. However, during our development of the search strategy we found significant overlap between PubMed and other databases (eg, CINAHL). Finally, selection criteria were rigorous; therefore, some relevant papers may have been excluded. Inclusion and exclusion criteria were determined through discussions by study authors, by reviewing best practices in scoping reviews. Search terms and inclusion criteria were designed to address our conceptualization of health information technology and community engagement previously described. However, other conceptualizations (eg, health information technology tools for tracking data of community members without collaborative development or data collection) are possible and could be explored in future studies.

### Conclusions

This study addresses the gap in the literature regarding the extent to which health information technology and community-engaged research are utilized concurrently, types of health information technology tools used, the various community engagement strategies utilized, and key outcomes assessed. Our results create a current assessment of the literature from which to promote further ideas for expanding the breadth and depth of technology utilization along with community and stakeholder engagement in health-related research to create more relevant and culturally centered outcomes and health information technology tools.

Furthermore, this scoping review showcases the types of health information technology interventions and tools that are currently utilized and presents successful examples of community engagement across the health information technology research spectrum. Thus, it provides researchers with starting points for expanding community engagement and the use of health information technology in health-related research. Many studies included in this review highlight the opportunity and efficacy of community engagement to drive change and to ensure sustainability. Utilization of health information technology offers great potential for promoting diverse stakeholder engagement and for establishing long-term and equitable research partnerships.

## Abbreviations

PCORI: Patient-Centered Outcomes Research Institute
